# Missing Measurements of Sesquiterpene Ozonolysis Rates and Composition Limit Understanding of Atmospheric Reactivity

**DOI:** 10.1021/acs.est.3c10348

**Published:** 2024-04-26

**Authors:** Gabriel Isaacman-VanWertz, Graham Frazier, Jeff Willison, Celia Faiola

**Affiliations:** † Charles E. Via, Jr. Department of Civil and Environmental Engineering, Virginia Tech, Blacksburg, Virginia 24061, United States; ‡ U.S. Environmental Protection Agency, Research Triangle Park, Durham, North Carolina 27709, United States; § Department of Ecology and Evolutionary Biology, University of California Irvine, Irvine, California 92697-2525, United States; ∥ Department of Chemistry, University of California Irvine, Irvine, California 92697-2525, United States

**Keywords:** atmospheric chemistry, biogenic volatile organic compounds, photochemistry, ozonolysis, monoterpene

## Abstract

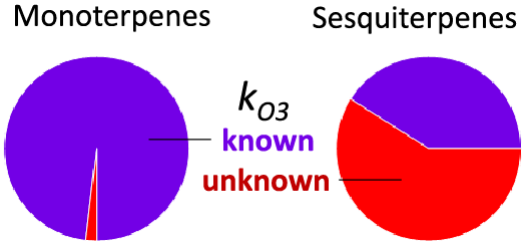

Emissions of biogenic reactive carbon significantly influence atmospheric chemistry, contributing to the formation and destruction of secondary pollutants, such as secondary organic aerosol and ozone. While isoprene and monoterpenes are a major fraction of emissions and have been extensively studied, substantially less is known about the atmospheric impacts of higher-molecular-weight terpenes such as sesquiterpenes. In particular, sesquiterpenes have been proposed to play a significant role in ozone chemical loss due to the very high ozone reaction rates of certain isomers. However, relatively little data are available on the isomer-resolved composition of this compound class or its role in ozone chemistry. This study examines the chemical diversity of sesquiterpenes and availability of ozone reaction rate constants to evaluate the current understanding of their ozone reactivity. Sesquiterpenes are found to be highly diverse, with 72 different isomers reported and relatively few isomers that contribute a large mass fraction across all studies. For the small number of isomers with known ozone reaction rates, estimated rates may be 25 times higher or lower than measurements, indicating that estimated reaction rates are highly uncertain. Isomers with known ozone reaction rates make up approximately half of the mass of sesquiterpenes in concentration and emission measurements. Consequently, the current state of the knowledge suggests that the total ozone reactivity of sesquiterpenes cannot be quantified without very high uncertainty, even if isomer-resolved composition is known. These results are in contrast to monoterpenes, which are less diverse and for which ozone reaction rates are well-known, and in contrast to hydroxyl reactivity of monoterpenes and sesquiterpenes, for which reaction rates can be reasonably well estimated. Improved measurements of a relatively small number of sesquiterpene isomers would reduce uncertainties and improve our understanding of their role in regional and global ozone chemistry.

## Abstract 2

Plant emissions play a large role in ozone formation and destruction. This study examines the complexity of sesquiterpenes, a major class of emissions, and finds that little is known about some of its chemical reactivity.

## Introduction

Emissions of reactive carbon to the atmosphere drive atmospheric chemical reactions that form and destroy important secondary pollutants, particularly secondary organic aerosol (SOA) and ozone (O_3_).^1^ These pollutants are major global drivers in loss of human life, damage to ecosystems, and decreases in global crop production, as well as serving as cloud condensation nuclei that play an important role in controlling cloud formation that strongly impacts albedo the Earth’s energy balance.^2−7^ Globally, the significant majority of this reactive carbon is emitted by the biosphere^8,9^ for ecological and biological reasons ranging from predation mitigation to thermal control to communication between organisms.^10−15^ While most biogenic reactive carbon is emitted as isoprene and monoterpenes, a nonnegligible fraction (a model estimate of ∼30%^9^) is emitted as higher-molecular-weight terpene classes (e.g., sesquiterpenes and diterpenes), terpenoids, and a handful of well-studied small oxygenated compounds such as methylbutenol and methanol. Owing to their dominance and their ubiquity, a substantial body of research has been dedicated to understanding the atmospheric impacts of isoprene and monoterpenes, but substantially less is understood about other terpene classes. We focus here in particular on sesquiterpenes, which are modeled to account for only ∼3% of total emissions,^9^ but may have an outsize influence on atmospheric chemistry due to their relatively low volatility and high reported reaction rates.

Once emitted to the atmosphere, reactive organic carbon participates in atmospheric chemistry in a number of ways that are relevant to humans, ecosystems, and the climate. The impacts of reactive compounds can be approximately considered in terms of their contributions to atmospheric oxidant reactivity—that is, the chemical sink for major atmospheric oxidants such as ozone and hydroxyl (OH) and nitrate (NO_3_) radicals—and their aerosol yields.^16−19^ These parameters describe the extent to which secondary pollutants are formed, with the former quantifying the extent to which carbon participates in chemical reactions that form and destroy ozone and the latter quantifying the fraction of emitted mass that is expected to form particulate phase mass due to these reactions. These impacts are governed by the physicochemical properties of the reacting organic carbon, such as volatility and oxidation rate constants (k_OH_, , ). Unfortunately, physicochemical properties are highly dependent on molecular structure,^20^ so different isomers within a terpene class may have reaction rates or other properties that differ by orders of magnitude.

In biogenic-dominated environments, OH reactivity and SOA formation from direct biogenic emissions are dominated by isoprene and monoterpenes (and in some ecosystems, methylbutenol).^16,19,21^ In urban environments, volatile consumer products and vehicle emissions dominate.^22,23^ It is unlikely that larger terpenes such as sesquiterpenes or diterpenes contribute significantly to OH reactivity, as measurements in the southeastern United States (a hotspot of biogenic reactive carbon emissions) found closure within measurement error between top-down and bottom-up estimates of OH reactivity without including larger terpene classes.^16^ Research in a boreal forest has also found sesquiterpenes to contribute only a very small fraction of OH reactivity, and even then only for a short period of the year.^24^ Furthermore, the average OH reaction rate constants of the monoterpene and sesquiterpene mixtures in the southeastern United States are comparable,^25,26^ while sesquiterpenes are shown to be present at lower concentrations, so it is difficult to explain any major role they could play in OH reactivity. There are specific environments in which sesquiterpene emissions may contribute significantly or even dominate over other terpene emissions,^27−29^ where sesquiterpene contributions to OH reactivity and SOA formation are likely much more important, but measurements of ambient concentrations generally demonstrate low sesquiterpene concentrations. Similarly, several studies have found that SOA in the southeastern United States can be reasonably explained without accounting for a significant mass formed from sesquiterpenes.^30,31^ In contrast, some models have suggested significant fractions of SOA may come from sesquiterpenes, although these are known to have highly uncertain sesquiterpene SOA yields,^31−33^ and studies in other environments have observed short periods of high contributions from sesquiterpene SOA.^34^ This is possible in part due to the relatively high SOA yields of sesquiterpene compounds to lower-molecular-weight terpene classes^29,35,36^ and their efficient nucleation of new particles.^37^ Consequently, the role of sesquiterpenes in SOA formation is still uncertain, although a global major contribution does not seem to be consistent with current data.

Where there is still major uncertainty about the impact of sesquiterpenes is in terms of ozone reactivity. Studies in several environments (e.g., southeastern U.S., Amazon, southern Finland) have suggested that sesquiterpenes may play an outsize role in ozone chemical loss, due particularly to the very high ozone reaction rate constants of particular isomers such as β-caryophyllene.^24,26,38^ Unlike the case of OH reaction rate constants, average ozone reaction rate constants have been estimated to be an order of magnitude faster for an ambient mix of sesquiterpenes than for monoterpenes.^25,26^ Despite their lower concentrations, sesquiterpenes may consequently play a non-negligible role in ozone chemical loss in biogenic environments. For example, Yee and coworkers estimated that sesquiterpenes may account for one-half to two-thirds of ozone reactivity in the Amazon if the mass fraction of β-caryophyllene in essential oils from Amazonian tree species was representative of the composition of emissions,^38^ although the accuracy of that representativeness assumption is not well-known. Similarly, chemical destruction of ozone was found to be a dominant pathway for ozone loss in a forest in California, but could only be explained by the theoretical presence of biogenic emissions with very high ozone reaction rates,^39^ which could be sesquiterpenes. This possibility is supported by measurements in a boreal forest that found sesquiterpenes dominate ozone reactivity, primarily due to the major presence of β-caryophyllene.^24^ The role of sesquiterpenes on ozone reactivity in the atmosphere could be particularly important during periods of high emissions, which have been shown to increase during periods of herbivory^29,40^ or, in some cases, ozone-induced stress^41,42^ and might therefore reasonably be expected to increase due to climate-driven stressors. Similarly, mechanical damage and human activity can increase terpene emissions and change terpene composition,^26,43^ while different species of vegetation have different terpene ratios,^44^ so land use change will likely change impacts of sesquiterpenes on ozone budgets. Models struggle to predict observed year-to-year differences in ozone concentrations in forested regions,^45^ and significant uncertainty exists in the ozone budget.^46,47^ Given the large potential role, sesquiterpenes have been proposed to play in ozone chemical loss, a better quantitative understanding of their ozone reactivity is critical.

Unfortunately, truly constraining the impacts of sesquiterpenes on ozone reactivity and SOA formation is stymied in large part by two major gaps in the data. First, very few isomer-resolved measurements of sesquiterpenes are available, and even fewer that capture actual real-world concentrations,^24,26,38,48,49^ as opposed to emissions in a lab or greenhouse environment. Isomer-resolved measurements are critical, as ozone reaction rates can vary across 3 orders of magnitude ( = roughly 10^–17^ to 10^–14^ cm^3^ molec^–1^ s^–1^)^50^ so the impact of sesquiterpenes on ozone reactivity is strongly dependent on the specific isomer composition of the compound class. Studies have found β-caryophyllene to be a dominant source of ozone reactivity, yet reaction rates of other sesquiterpenes are mostly unknown.^50^ It is consequently critical to examine to what extent we understand the isomer-resolved composition of sesquiterpenes and to examine the extent to which we know ozone reaction rates and/or can trust our predictions of them.

In this work, we examine published data on sesquiterpene concentrations and emissions to assess the diversity of sesquiterpenes in order to broadly examine whether there is enough data to provide a general description of sesquiterpenes as a compound class. We further examine the body of available reaction rate constants to determine the extent to which estimations of ozone reactivity are reliable. We contrast the state of knowledge of sesquiterpenes and ozone reactivity with understanding of monoterpenes and of OH reactivity and determine that issues of uncertainty are unique to sesquiterpene ozone reactivity and likely do not impact many other aspects of terpene atmospheric chemistry. Lastly, we provide suggestions for specific compounds that may warrant deeper investigation by the atmospheric chemistry community.

## Methods

### Rate Constants

Measured rate constants are reported as compiled by McGillen and coauthors.^51^ For terpenes, the significant majority of OH and ozone reaction rates are from datasheets provided by the task group on Atmospheric Chemical Kinetic Data Evaluation of the International Union of Pure and Applied Chemistry (IUPAC).^50^ Rates are shown in figures as the recommended best estimate with uncertainties, as provided by the IUPAC task force. When no recommended best estimate is provided, the reaction rate in the figures is shown as only an error bar.

Estimated reaction rates are calculated by a structure–activity relationship, specifically that implemented by the AOPWIN module of the Estimation Programs Interface (EPI) Suite provided by the United States Environmental Protection Agency.^52^ This method uses the relationship published by Kwok and Atkinson.^53^ Molecular structures of terpenes are input as nonstereoisomeric SMILES strings (simplified molecular-input line-entry system).

### Terpene Composition

Isomer-resolved sesquiterpene composition was examined through two methods: global, annually average emissions using a common biogenic emission model, the Model of Emissions of Gases and Aerosols from Nature (MEGAN); and a compilation of published data on sesquiterpene emissions and concentrations.

MEGAN version 3.2^54^ was added to the Community Multiscale Air Quality (CMAQ) model^55,56^ and we run CMAQ as part of a coupled system with meteorological forcing from the Model for Predication Across Scales (MPAS) version 7.2.^57^ There are no simulated feedbacks between MEGAN and driving meteorology. The MPAS meteorological configuration includes modifications of Gilliam et al.,^58^ and we simulate a full year on a global domain with uniform 120-km horizontal resolution. We modify the MEGAN code to output the 147 species that are included in CMAQ’s CB6 chemical mechanism before the species are lumped together into 37 categories. The nonmeteorological inputs for MEGAN are available online (https://bai.ess.uci.edu/megan/data-and-code/megan32). For this study, we use leaf area index information from MPAS, growth form data set version 3a and the ecotype data set version 3b. We did not enable the drought stress option, which would have reduced emissions uniformly across species. Classification by terpene class follows classifications by Guenther and coauthors,^9^ solely including hydrocarbons in each chemical class and excluding related oxygenates (e.g., borneol is not included as a monoterpene, and nerolidol is not included as a sesquiterpene, though reported as related and grouped as such by Guenther and coauthors^9^). In measurements, a small number of sesquiterpenes are included that have the formula C_15_H_22_ instead of the more common C_15_H_24_, which have been reported in the literature as sesquiterpenoid (e.g., calamenene^38^) though are not included in MEGAN.

Published data were compiled using Web of Science search for defined terms. Data were included in the compilation provided it met the following criteria: measured either emissions or concentrations, was isomer-resolved, included greater than three isomers of sesquiterpenes, and made the manuscript data readily available either within the manuscript or in the Supporting Information. Most relevant was the term “sesquiterpene atmosphere,” which returned 116 results that capture most of the ambient concentration data reported in this work. This search term was selected as related terms such as “sesquiterpene concentration” return large numbers of results that do not pertain directly to the atmosphere by measuring either emissions or concentrations (e.g., published work on leaf storage or the content of essentially oils). However, as known relevant works were not captured by this search term, additional specific terms were used including “(sesquiterpene OR sesquiterpenes) AND speciated,” which provided 2 relevant works, and “(sesquiterpene OR sesquiterpenes) AND chromatogram,” which returned a small number of relevant works and mostly work on essential oils or other extractions. Lastly, the broad term “(sesquiterpene OR sesquiterpenes) AND (emission OR emissions)” was used and limited to manuscripts in the last 5 years (2018–2023) due to the large number of works captured by this term, returning 358 works. The substantial majority did not meet the criteria, but many emission works of specific species or environments were captured by this term. Manuscripts were excluded if included in a review of sesquiterpene emission data by Duhl and coauthors^59^ as these papers were used to create the emission factors used in the MEGAN emission model and are thus approximately captured by the global MEGAN emission estimates shown.

References included in the compilation of data presented in this work include concentration and emission measurements of ecosystems or prevalent forest vegetation. This work does not include bodies of literature on the emissions of specific cultivated crops^60−64^ or on aromas or headspace of specific flowers or ornamental plants.^65−69^ Any works captured by these search terms in these bodies of literature are included in the references in the prior statement but not compiled or discussed further in the present work, although a cursory review suggests the complexity and variability of the chemical class are similar in these works as that will be shown in the present work.

Concentrations are measured in a pine forest in California by Bouvier-Brown et al.^48^ and in Colorado by Chan et al.;^70^ in boreal forests by Hakola et al.,^49^ Hellén et al.,^24^ and Vestenius et al.;^71^ in the Amazon by Yee et al.;^38^ in a chamber with Scots pine separated into healthy and herbivory-stressed trees by Faiola et al.;^40^ in the South African Savannah by Jaars et al.;^72^ and in the southeastern U.S. at both a forest and a farm by Frazier et al.^26^ Emissions are measured from soil chambers at two sites in the Amazon by Bourtsoukidis et al.;^73^ a boreal forest floor by Mäki et al.^74^ and Wang et al.;^75^ wetlands by Hellén et al.;^27^ Scots pine by Ylisirniö, et al.^76^ and separated into healthy and herbivory-stressed by Joutsensaari et al.,^77^ Faiola et al.,^29^ and Kivimäenpää et al.;^78^ downy birch by Hellén et al.;^28^ Norway spruce by Thomas et al.;^79^ a mixture of subtropical trees found in China by Zeng et al.;^80^ diseased balsam popular by Jiang et al.;^81^ healthy and herbivory-stressed mountain birch by Ryde et al.;^82^ healthy and ozone-stressed Canary pine by Vo and Faiola^83^ and the Brazilian tree *Croton floribundus* by Bison et al.;^41^ Mediterranean vegetation cork oak and the shrub labdanum by Haberstroh et al.;^84^ a mixture of shrubs found in China by Zhang et al.;^85^ several deciduous tundra shrubs by Simin et al.;^86^ and healthy and heat- and herbivory-stressed tundra by Ghimire et al.^87^ In all, a total of 27 manuscripts are compiled in this work, including 9 measuring concentrations and 18 measuring direct emissions.

Isomer-resolved monoterpene composition was compiled using a Web of Science search for the analogous term “monoterpene atmosphere,” which returned 517 results. Compiled data are not intended to be comprehensive but rather illustrative in contrast to data on sesquiterpenes. References used in this sample include measurements of concentrations in the forest of Borneo by Jones et al.;^88^ a boreal forest by Hakola et al.;^49^ a hemiboreal forest by Noe et al.;^89^ the Amazon by Maria Yáñez-Serrano et al.;^90^ and in the southeastern U.S. by McGlynn et al.^25^ A review by Geron et al.^44^ compiles emission data for monoterpenes and was one of several references incorporated into the MEGAN emission model and is thus represented in the global annual emissions estimated by MEGAN here. References included for monoterpenes do not include stress-driven emissions because: changes in stress-driven changes in monoterpenes emissions are highly variable,^91^ monoterpenes are not the primary focus of the present work, and (as is discussed below) most reported stress-impacted monoterpenes have known ozone reaction rates so do not change the general conclusions of this work.

## Results and Discussion

**Figure 1:**
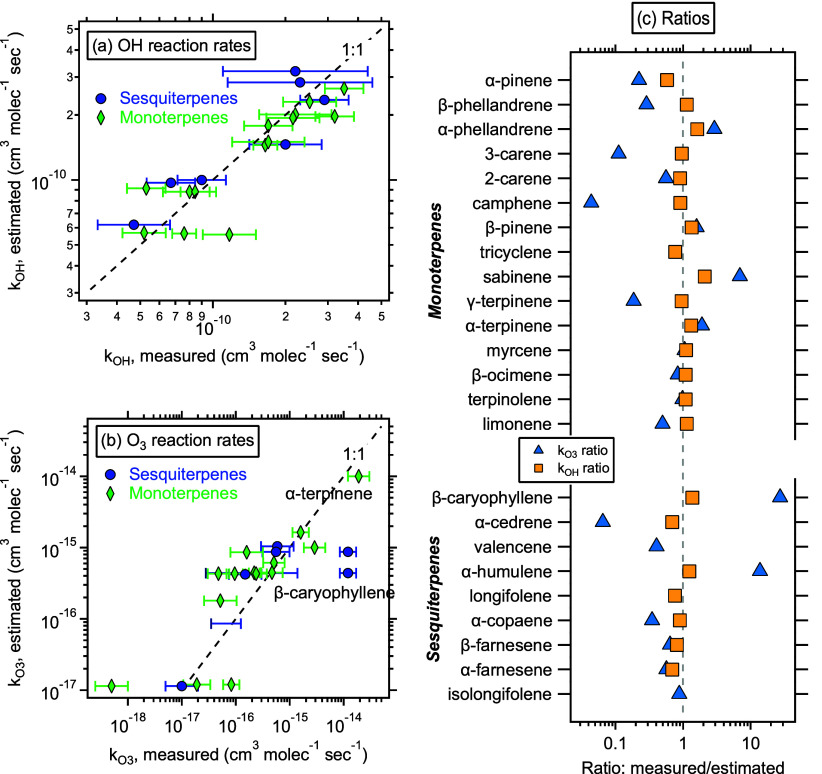
Comparison between measured reaction rate constants and those estimated by structure–activity relationships for monoterpenes and sesquiterpenes. Second order reaction rate constants are shown for terpenes with (a) a hydroxyl radical and (b) ozone. Best estimates are included as markers with error bars for uncertainty; where no best estimate is provided in the literature, the error bar represents the range of reported values. (c) The ratio of measured to estimated reaction rate constants for both the compound class and both oxidants. Dashed lines show where measured and estimated rate constants are different.

Reaction rates for monoterpenes and sesquiterpenes generally fall within the same quantitative ranges at typical ambient temperatures. While measured OH reaction rates span approximately 1 order of magnitude, from roughly 5 × 10^–11^ to 4 × 10^–10^ cm^3^ molec^–1^ s^–1^, ozone reaction rates span 3 orders of magnitude, from 1 × 10^–17^ to 2 × 10^–14^ cm^3^ molec^–1^ s^–1^ (Figure 1a,b). For context, this suggests that under general ambient conditions ([OH] = 2 × 10^6^ molecules cm^–3^, [O_3_] = 40 ppb), OH reactions for nearly all terpenes occur on time scales on the order of an hour, while lifetimes for ozone reactions may be anywhere from faster than a minute to longer than a day. Consequently, the isomer-resolved understanding of the mixture is much more important for ozone reactions than for OH reactions. The distribution of reaction rates is similar for each compound class, supporting previous conclusions that the ambient composition-weighted average OH reaction rate constant is only ∼50% higher for sesquiterpenes (1.1 × 10^–10^ cm^3^ molec^–1^ s^–1^) than for monoterpenes (6.9 × 10^–11^ cm^3^ molec^–1^ s^–^1 ). Together these data suggest that OH reactivity of terpenes will generally scale with the total concentration with little impact of isomer composition, and the importance of sesquiterpenes in an environment will be roughly proportional to their concentrations relative to monoterpenes. Sesquiterpenes would be expected to be important for OH reactivity only in high-sesquiterpene environments.

In contrast, ozone reaction rates are far more dependent on the isomer composition of the mixture. While the fastest measured reaction rate is actually a monoterpene, α-terpinene, this isomer is generally not a major contributor to the monoterpene class. The high measured reaction rates of β-caryophyllene and α-humulene, which are found in sesquiterpene mixtures, have led to previous conclusions that these specific isomers play a significant role in ozone chemical loss. For the same reason, the ambient concentration weighted average ozone reaction rate constant of the sesquiterpene mixture has been reported as more than an order of magnitude faster than that of monoterpenes. However, it is also clear that the fast reaction rates of β-caryophyllene and α-humulene are significantly higher than those predicted by structure activity rates.

While OH reaction rates estimated by structure–activity relationships are within or near the uncertainty of the measured rate, the same is not true for ozone reaction rates. The ratio of measured to estimated reaction rates (Figure 1c) is nearly always near unity for OH reaction rates of both sesquiterpenes and monoterpenes. The skill of the estimation approach for OH is due, in part, to the relatively low range of OH reaction rate constants (in other words, it is easy to predict a number that is not highly variable). In contrast, ozone reaction rate constants differ from estimates by up to a factor of roughly 25 times higher (β-caryophyllene) to roughly 20 times lower (α-cedrene, camphene). Considering the range of ozone reaction rates and the relative imprecision with which they can be predicted, it is clear that an accurate quantification of the ozone reactivity of a mixture requires knowledge of both the isomer composition of a terpene class (be it monoterpene or sesquiterpene) and measured (not estimated) ozone reaction rate constants. How well then do we know the typical composition of the monoterpene or sesquiterpene class, and for what fraction of each class do we know the ozone reaction rate?

**Figure 2:**
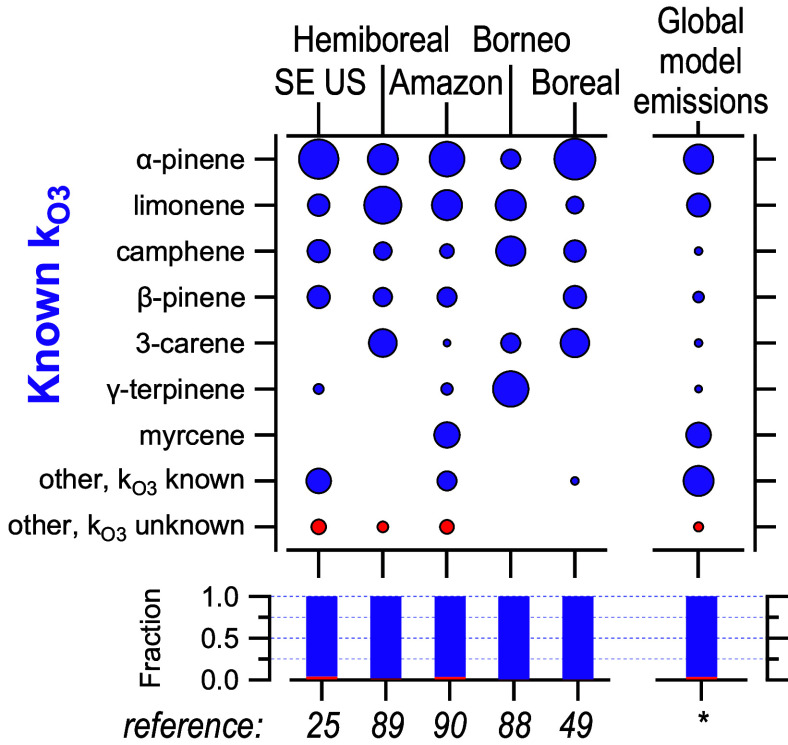
Composition of monoterpenes reported in a selection of published works, divided into isomers with known (purple) and unknown (red) ozone reaction rate constants. Area of the circle corresponds to mass fraction of each isomer. Bottom panel shows fraction for which ozone reaction rate constant is known. Asterisk is global annual model emission data from this work. References included are labeled on the top with a very brief description of the measurement location and numbered by their reference number at the bottom. Data are provided as Table S1.

A significant fraction of monoterpene mass in reported concentration measurements is composed of only a few isomers (Figure 2), although 22 different monoterpenes are estimated in the MEGAN emission model. More than half the reported mass of monoterpenes can generally be accounted for by only α-pinene, β-pinene, limonene, and camphene, with a few others often making up most of the balance (γ-terpinene, myrcene, 3-carene). These are broadly also the isomers reported by Geron and coauthors^44^ in their review of monoterpene emissions from a wide range of vegetation. Ratios vary somewhat, so a single mixture may not be representative of the globe, but generally a mixture representative of monoterpenes contains a large fraction of α-pinene, roughly one-third as much β-pinene, and some large component of limonene (often, but not always, lower than that of pinenes). It should be noted, however, that the ratio of pinenes to limonene and myrcene is a critical feature in understanding the ozone reactivity of the monoterpene mixture due to the fast reaction rates of the latter. Nevertheless, the composition weighted average ozone reaction rate constant is typically within approximately 50% of the α-pinene reaction rate. Most importantly, more than 96% of the monoterpene mass (comprising 15 of the 22 reported isomers) has a known ozone reaction rate. Though stress-driven emissions are not included in this compilation, it is worth noting that most of the isomers observed to change with stress also have a known ozone reaction rate.^91^ These data suggest that impacts of monoterpenes on ozone reactivity can be quantified with low uncertainty provided isomer-resolved measurement. Even in the absence of isomer-resolved data, ozone reactivity of monoterpenes estimated using α-pinene as a surrogate is likely accurate within a factor of 2 or better.

**Figure 3:**
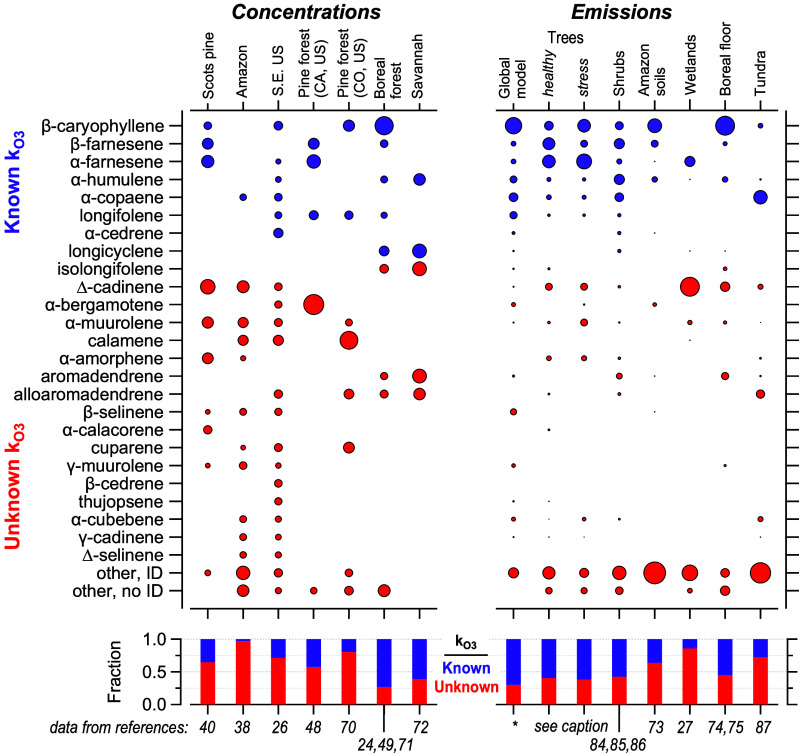
Composition of sesquiterpenes reported in published work, divided into isomers with known (purple) and unknown (red) ozone reaction rate constants. Area of the circle corresponds to mass fraction of each isomer. Bottom panel shows fraction for which ozone reaction rate constant is known. Asterisk is global annual model emission data from this work. Measurements are separated into those that report concentrations in a given environment and those that report emissions from a certain source. Compiled data are categorized for legibility and provided as Table S3; for complete data see Table S2. References included are labeled on the top with a very brief description of the measurement location and numbered by their reference number at the bottom. For trees, reference numbers include healthy trees from refs (28), (29), (41), (76)−^80^, (82), (83), and (84) and stressed trees from refs (29), (41), (77), (78), (80)−^83^, and (84).

In contrast to monoterpenes, sesquiterpenes are found to be substantially more diverse and far more poorly characterized. Across the literature, 72 identified sesquiterpenes are reported in ambient atmospheres or emissions, with a dozen more reported with no definitive identification (and thus may or may not represent the same isomers across studies). Of these isomers, only 9 have known ozone reaction rates, and these typically account for approximately half (range: 3% to 99%) of the measured sesquiterpene mass (Figure 3). In Figure 3, the 27 compiled manuscripts are grouped into 14 categories (plus the model) to improve legibility; the full compositional data are available as Table S2. Furthermore, the dominant isomer is highly variable. While β-caryophyllene is frequently observed and is sometimes dominant, farnesene, cadinenes, and muurolenes are also frequently dominant or significant and the latter two classes have no known ozone reaction rates. There is also some trend toward higher fractions of unknown mass as the number of measured isomers increases; studies that measured more than 10 isomers find on average that two-thirds of the sesquiterpene mass does not have a known ozone reaction rate, compared to an average 40% unknown mass in studies with 10 or fewer isomers (different at the *p* < 0.01 level using Wilcoxon–Mann–Whitney test). Notably, the global emission model estimates that 70% of the sesquiterpene mass is comprised of isomers for which ozone reaction rates are known, which is not in line with most of the measurements. This fraction of known mass is particularly high considering the large number of species reported by the model (35 isomers), considering that all studies with higher than 70% known mass measured fewer than 10 isomers. Many of the papers reviewed by Duhl and coauthors^59^ and then used to estimate sesquiterpene emissions in MEGAN report only a small number of isomers (1 to 10, those with 3 or more included in Table S2), so may be biased toward better-studied or more ubiquitous compounds.

The diversity of the sesquiterpene class can be quantified as an effective species number that describes a theoretical number of species needed to describe the distribution of sesquiterpenes (alpha diversity, *D*_α_ = e^*H*α^, where *H*_*α*_ is Shannon entropy, the negative sum-product of the mass fraction and the natural log of the mass fraction of each sesquiterpene isomer).^92^ When all isomers are present in the same concentration, the effective species number is equal to the number of reported species, while a low ratio of effective species number to measured species indicates the dominance of one or a small number of isomers. The effective species number of sesquiterpenes increases with the number of measured isomers and is as high as 16.4 (southeastern U.S. forest). The effective species number is on average one-half (for emissions) to three-quarters (for concentrations) of the number of measured isomers, indicating that there is generally no dominant isomer(s) and comprehensive isomer-resolved composition of sesquiterpenes is necessary to understand the compound class. In contrast, the effective species number of measured monoterpenes is only as high as 6.0, and monoterpenes can be mostly almost entirely described by a set of 7–10 isomers. It is therefore not clear whether there is a consistent surrogate or mixture that can be used to approximate the impacts of the sesquiterpene class without isomer-resolved data. Furthermore, even with isomer-resolved data, the lack of known ozone reaction rates suggests that the ozone reactivity of the compound class cannot be reliably estimated.

There are a few qualitative trends that can provide some limited guidance. For example, β-caryophyllene tends to be more present in emission data and is less important in concentration data, consistent with its fast reaction rate, leading to high oxidative loss. Emissions from sources other than trees or shrubs, such as soils and forest floor, may be dominated by isomers rarely reported in the atmosphere, such as α- and γ-gurjunene and α-himachalene. This may imply similarly fast reaction rates or may be due simply to a low influence of these emission sources on ambient atmospheres. In ambient measurements, the sesquiterpene mixture is likely to contain moderate concentrations of Δ-cadinene, α-muurolene, α-amorphene, and alloaromadendrene.

## Implications

The sparse data on sesquiterpenes and their ozone reaction rates suggest that there is currently insufficient information about the chemical class to quantify their impact on the ozone budget. Sesquiterpenes are estimated to be less efficient at increasing ozone and are treated in some models as reacting with ozone much faster than other terpenoids.^93,94^ In reality, these properties are isomer dependent and are not well constrained by measurements. Information about other important properties, such as the efficiency with which they form organic nitrogen compounds that can transport ozone chemistry, are mostly limited to studies of nitrate radical chemistry on a few isomers.^95,96^ Consequently, the role of sesquiterpenes in the formation, destruction, and spatial distribution of ozone is not well understood. Given these uncertainties in the ozone budget, the lack of model skill in predicting variability in ozone, and limited studies that have suggested a major role for sesquiterpenes in ozone chemical loss, it is important to fill our gaps in sesquiterpene data and reaction rates.

This work is not intended to suggest that sesquiterpenes are necessarily major contributors to global ozone budgets but rather illuminates that there is simply insufficient data to confidently assess whether they are. If, for example, the true ozone reaction rate of Δ-cadinene is 25 times higher than estimated (as is the case for β-caryophyllene), that would substantially increase the impact of sesquiterpenes on ozone reactivity. Like β-caryophyllene, Δ-cadinene is frequently present in emissions, though unlike β-caryophyllene, it is also present in moderately high concentrations. So perhaps an unexpectedly high reaction rate is not likely, but there is currently no way to know. Until a much larger fraction of sesquiterpenes have known ozone reaction rates and until a more comprehensive understanding of isomer-resolved sesquiterpene composition is available, the impact of this compound class on ozone chemical loss can only be estimated with very high uncertainty. It is currently not feasible to estimate a realistic average ozone reaction rate for this chemical class, nor provide a reliable surrogate, despite previous efforts to do so (including work by some these authors^26^). However, it is likely reasonable to assume that β-caryophyllene is not such a surrogate, as its ozone reaction rate is substantially higher than that of most other sesquiterpenes with known rates, and a best estimate of average ozone reaction rate for the sesquiterpene class is likely several times or an order of magnitude lower than that of β-caryophyllene.

The compiled data on sesquiterpene isomers suggest a small number of isomers that are likely the most important targets for future measurements of ozone reaction rate constants. The isomers Δ-cadinene, α-muurolene, and alloaromadendrene are present in most measurements, often at high levels in ambient concentrations. Similarly, α-gurjunene and α-himachalene dominate emissions from some measured nonvegetation sources, and α-amorphene is observed at high levels from Scots pine. Known reaction rates for only these 6 isomers would bring the fraction of identified mass with known ozone reactivity up to more than half for nearly all studies and two-thirds or more for a majority of studies. With data for only a few additional isomers (calamene, α-bergamotene, and β-cubebene), more than three-quarters of identified sesquiterpene mass would have a known ozone reaction rate in a majority of studies. This task is made difficult by a number of systems with unique dominant isomers, for example, the significant contribution to mountain birch emissions by dihydro-neoclovene, which is not reported in any other system, as well as the large fractions of unidentified sesquiterpene mass in many emission measurements. Consequently, a small number of known ozone reaction rates cannot bridge the gap to fully known ozone reactivity of sesquiterpenes due to the sheer diversity of the chemical class, but these data would significantly reduce the uncertainty. Given the potentially large role of sesquiterpenes in ozone reactivity, improved characterization of not only the isomer composition but also chemical kinetics studies on their reaction rates is a critical research task for the community if we are to better understand ozone chemical sinks and loss processes.

## Supporting Information Available

Tables S1–S3 containing mass fraction of monoterpenes and sesquiterpenes used in Figures 2 and 3 and sesquiterpene mass fractions are also included for studies included in the review by Duhl and coauthors,^59^ excluding studies with fewer than three isomers reported (XLSX)
